# A Review on the Effect of High Pressure Processing (HPP) on Gelatinization and Infusion of Nutrients

**DOI:** 10.3390/molecules25102369

**Published:** 2020-05-20

**Authors:** Akash Kaushal Balakrishna, Md Abdul Wazed, Mohammed Farid

**Affiliations:** Department of Chemical and Materials Engineering, University of Auckland, Private Bag 92019, Auckland 1142, New Zealand; amag410@aucklanduni.ac.nz (A.K.B.); mwaz610@aucklanduni.ac.nz (M.A.W.)

**Keywords:** high pressure processing (HPP), gelatinization, infusion, quality improvement

## Abstract

High pressure processing (HPP) is a novel technology that involves subjecting foods to high hydrostatic pressures of the order of 100–600 MPa. This technology has been proven successful for inactivation of numerous microorganisms, spores and enzymes in foods, leading to increased shelf life. HPP is not limited to cold pasteurization, but has many other applications. The focus of this paper is to explore other applications of HPP, such as gelatinization, forced water absorption and infusion of nutrients. The use of high pressure in producing cold gelatinizing effects, imparting unique properties to food and improving food quality will be also discussed, highlighting the latest published studies and the innovative methods adopted.

## 1. Introduction

**Figure 1:**
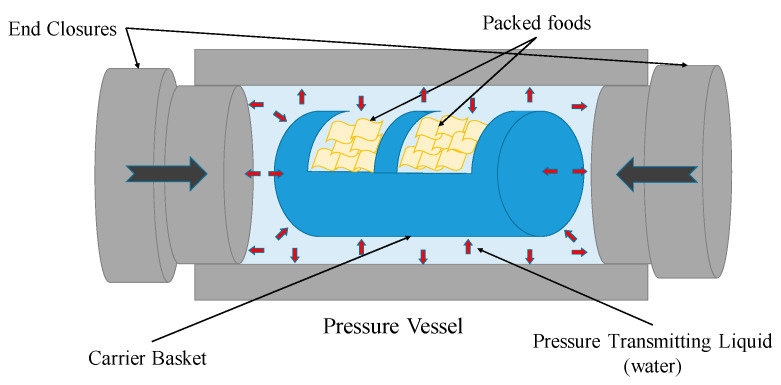
Schematic representation of a high pressure processing (HPP) vessel.

High pressure processing (HPP) or high hydrostatic pressure (HHP) are terms that describe subjecting foods to pressures in the order of thousands of atmospheric pressures. The foods are usually vacuum packed and placed into a basket, which is then loaded into the HPP machine, as shown in Figure 1.

The two openings are closed with end closures and a pressure transmitting liquid, which is usually water, is used to pressurize the vessel. The combined effect of a high pressure pump(s) with a pressure intensifier(s) is used to generate the required pressure. The foods are then held at a particular pressure for a specified duration. This is followed by a quick decompression and unloading. Under compression, there is an increase in water temperature of up to 3 °C for every 100 MPa increase in pressure. Lipid based foods experience a slightly higher increase in temperature with pressure [1]. The temperature returns to close to the initial temperature upon depressurization. HPP has been widely investigated for increasing the shelf life of foods through the inactivation of microorganisms [2], spores [3] and enzymes [4] present in foods. HPP can also be used for functional and physical modifications of foods [5,6]. It has also been tested for creating pressure-shift freezing and thawing, which is outside of the scope of this review. This review covers the application of HPP for inducing low temperature gelatinization, changing food properties and enhancing food quality. Some of the prior review works have been exclusively done on high pressure gelatinization of pure starch [7,8] and on high pressure infusions [9]. This article is a unique review of the latest developments in these fields and includes discussions on other aspects of gelatinization of starch in foods, gelatinization in other compounds, texture and property enhancement in whole foods, nutritional, flavour, aroma related infusions, etc.

## 2. High Pressure Gelatinization

### 2.1. Starch Gelatinization

**Figure 2:**
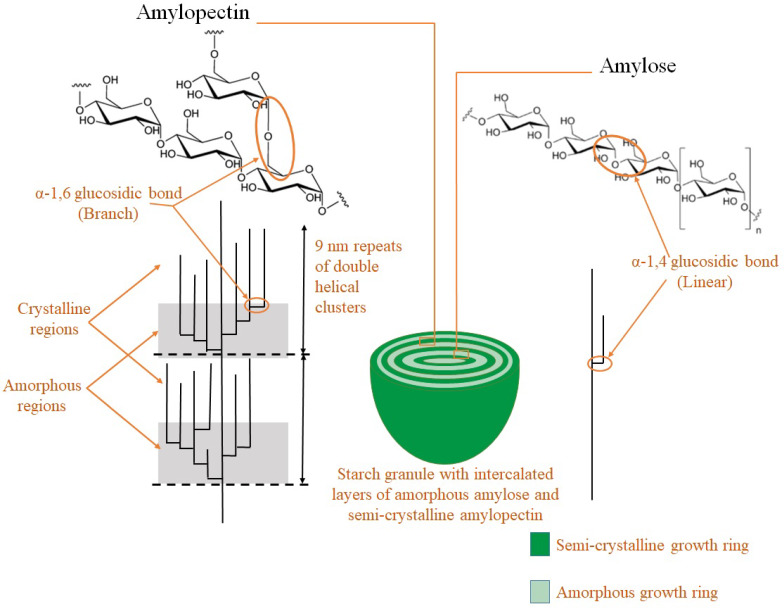
Schematic representation of a starch granule with its structure and types (redrawn from O’Neill and Field [11] and Raguin and Ebenhöh, 2017 [12]).

Starch is a polymeric carbohydrate that consists of two types of molecules, amylose and amylopectin (Figure 2). When starch granules swell up in the presence of water and heat, the process is called gelatinization. During this process, water molecules are adsorbed onto the amylose and amylopectin molecules, breaking the intermolecular hydrogen bonds present in starch. On cooling, starch loses water and reformation of hydrogen bonds takes place; this is called retrogradation. High pressure processing causes cold gelatinization of starches. Pea starch gelatinizes at 25 °C and pressures above 400 MPa. The presence of water is necessary, without which there will be no effect of pressure on starch [10].

#### 2.1.1. Gelatinization Mechanism

**Figure 3:**
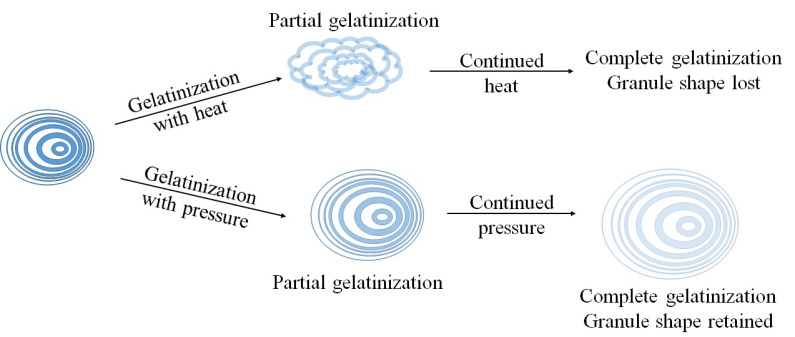
Schematic representation of heat and pressure gelatinization (redrawn from Yamamoto et al., 2009 [15]).

Previous studies have suggested that the use of high pressure results in lowering the gelatinizing temperature of the starch and that the gelatinization temperature is a non-linear function of pressure [13,14]. The higher the pressure, the greater the reduction is. It has been reported that the mechanisms of gelatinization in the pressure treated starch and heat treated starch are different [15]. Starch, when heated with water, swells up and loses the ellipsoidal lamellar ring structure along with its crystallinity; whereas pressure gelatinization maintains the shape and structure of the granules and lamellae as shown in Figure 3. Similar conclusions were reported in the study comparing thermal gelatinization with pressure gelatinization of wheat starch [16]. The SEM analysis of the starch granules gelatinized using heat and pressure revealed that the gelatinization caused by heat results in disruption (structure loss) of the granules, whereas the pressure treated ones undergo distortion (structure change). Heat treatment acted by gelatinizing only the less stable crystallites, whereas the pressure treatment acted on both the less stable and more stable crystallites.

Another study showed that gelatinization occurs at different pressures and temperatures for the different starches [17]. Potato starch is highly resistant to pressure, whereas that of wheat is much more sensitive. The degree of gelatinization increases with temperature at any pressure and vice versa and the action caused by pressure is time-dependent.

#### 2.1.2. Properties of Pressure Gelatinized Starch

High pressure treatment of starch results in starch with unique properties. Along with the preservation of the granule structure, the starch gel formed by HPP treatment of tapioca starch showed greater hardness [18]. When tapioca starch was pressurized at 600 MPa in the temperature range of 30 °C to 80 °C, the hardness was more evident in the gels treated at lower temperature and became less prominent with the increase in temperature. At high pressure, treatment time decreased the hardness of gels, but the effect was very small and became evident only after long processing times of more than 30 min. The starch–starch and starch–water interactions in the HPP induced gel were much higher. Post storage analyses of the gels were conducted after a storage time of 28 days under storage conditions of refrigeration (4 °C) and freezing (−18 °C). In terms of textural change and amylopectin recrystallization, the HPP induced starch gels showed better stability after storage compared to their thermally treated counterparts. The properties of wheat starch treated with HPP were similar. HPP produced denser gels, and these gels had a lower grade of retrogradation when compared to the heat treated gel [19]. Starch granule preservation, limited granule expansion and low amylose release were other important properties observed in HPP treated granules. Another study on wheat starch showed that the pressure treated wheat starch had properties of higher hardness, gumminess and chewiness up to a gelatinization percentage of about 40%, after which these properties were greater in the heat treated starch [16]. Colussi et al. [20] examined the effect of cyclic pressure treatments on potato starch gels. The SEM analysis revealed that cyclic HPP caused eruptions (bursting of granules), abrasions (surface deformities) and disruption of starch granules with varying severity of treatments. The gel structure became more compact with smaller spaces within fragment networks after HPP. The pressure treated samples after a seven-day retrogradation period showed a further increase in the density and compactness.

**Figure 4:**
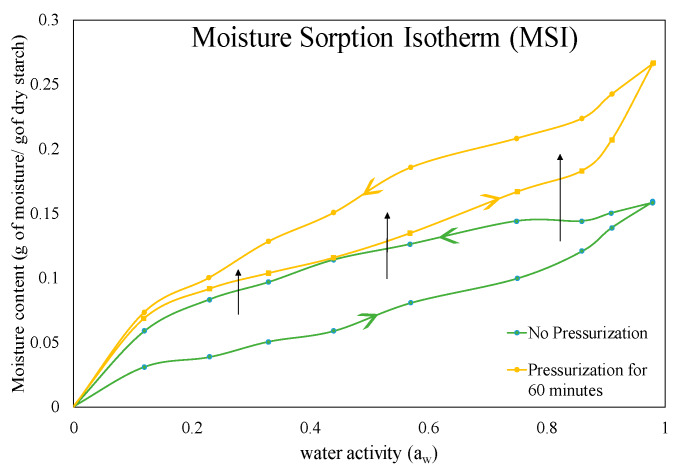
Change in the hysteresis of starch observed after high pressure treatment (plotted using the data from Santos et al., 2014 [21]).

Use of HPP on maize starch led to an increase in moisture content for a given water activity with pressurization time following an adsorption-desorption isotherm [21]. The effect of the shift in the water sorption isotherm was higher for both higher water activity and lower water activity (Figure 4). The shift was also correlated to pressurization time; and longer treatment time led to a higher shift. The effect of high pressure treatment time from 5 to 60 min also led to a 2.7 fold increase in water-holding capacity of maize starch. The extent of starch hysteresis observed after the HPP treatment was lower (depending on the treatment time) until the water activity reached 0.44. Subsequently, the degree of hysteresis in pressure treated starch became much higher in comparison to unpressurized starch.

Waxy rice, which has no amylose content, showed structural disruption and granular structure disappearance at very high pressures of 600 MPa. HPP increased the lamellar repeat distance proportional to pressure [22]. The relative crystallinity decreased as the pressure increased from 100 MPa to 600 MPa. Starches can be highly resistant to pressure. Chestnut flour suspensions did not completely gelatinize in the range of 400 MPa to 600 MPa [23]. There was no change in granule particle size or hydration properties. X-ray diffraction diagrams given by native starches are mainly of two types [24]. These are the A type, generally exhibited by cereal starches and the B type, usually shown by tubers and high amylose starches. C type diffraction pattern, which is a combination of A type and B type diagrams, is typical for legume starches. The V form crystalline complex rarely seen in native starches is observed when there is a complex formation of amylose with fatty acids and monoglycerides. Sorghum starch did not show any change in the crystallinity from the native starch up to 480 MPa and continued to show the A type crystalline pattern [25]. The starch underwent gelatinization at 600 MPa and then showed B type crystalline pattern. Properties such as water absorption capacity and thermal stability increased, whereas oil absorption capacity, swelling power and viscosity decreased with increasing pressure levels.

A non-conventional starch extracted from the seeds of mangoes and referred to as mango kernel starch (MKS) was investigated for its changes on treatment with HPP [26]. The HPP treated MKS had stronger starch aggregations and lower retrogradation tendencies. The SEM images of MKS granules, when treated with high pressure, showed hardly any structural changes, but the surface of the starch granules lost its smoothness and became rough.

A summary of the highlights of the studies on the properties of starches can be seen in Table 1.

#### 2.1.3. Influence of Additives on Gelatinization

When gelatinization of maize starch was studied in the presence of hydrocolloids, it was found that the hydrocolloids ensure the availability of water to the starch granules, but some hydrocolloids, like xanthan gum, can result in lower levels of gelatinization [28]. The reason could be the shielding effect provided by the hydrocolloids at high pressure. Teixeira et al. [28] also reported that high pressure changes the maize starch crystal from the A type XRD pattern to give patterns with characteristics of both A type and B type starch. The extremely high pressure of 700 MPa, long time of pressure treatment or the presence of hydrocolloids favoured a V-crystalline complex. The presence of NaCl premixed in corn starch solutions after high pressure treatment did not affect the granular structure of the starch, but it decreased the stability of the emulsion of solutions prepared with it [29]. A study on the influence of pH and osmolarity (of salts) in HPP gelatinization of waxy starches of rice and corn revealed that the rate of gelatinization and its extent decreased by increasing the osmolarity of the salts, whereas the influence of pH was minor [30]. Simonin et al. [30] further stated that the reason behind this behavior was explained by the Hofmeister series; thus: “Anions coupled with constant cations increase the solubility of protein or decrease the starch gelatinization temperature in the order SO_4_^−^ < CH_3_COO^−^ < Cl^−^ < Br^−^ < NO_3_^−^ < ClO_4_^−^ < I^−^ < SCN^−^.” Additionally, the salts used in the experiment were kosmotropes (salts that structure water around macromolecules and increase the viscosity of the solution), which meant the availability of the solution to the starch granules would be delayed. The gel strength and extent of swelling were found to be in direct correlation with the extent of gelatinization.

#### 2.1.4. Digestibility

**Figure 5:**
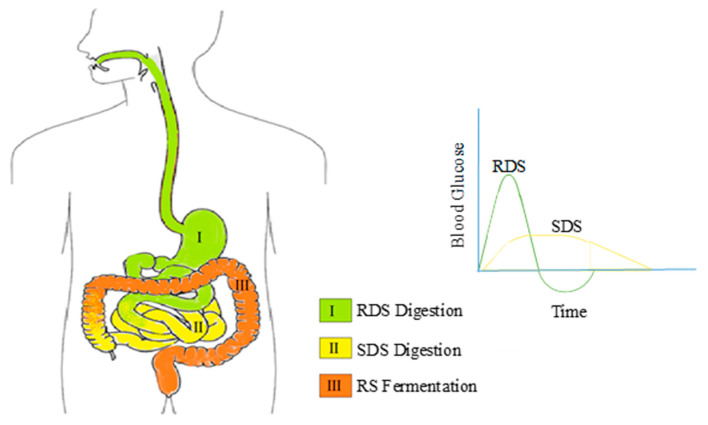
Digestion of different kinds of starches in the digestive system and their glycemic response in the blood glucose (redrawn from Miao et al., 2015 [34] and Sorndech et al., 2018 [35]).

The digestion of different components of starch takes place at a different rate in the human intestine [31]. The starch can thus be classified: rapidly digestible starch (RDS), slowly digestible starch (SDS) and resistant starch (RS) components. As the name suggests, RDS digests very quickly and SDS digests slowly in the small intestine (Figure 5). RS escapes digestion and undergoes fermentation in the large intestine. The pressure treated samples of starch have a higher digestibility if the pressure treatment of about 500 MPa is carried out at temperatures above 45 °C [32]. Subjecting starch to high pressure causes gelatinization but a long time of exposure to high pressure gives it a new form that is less digestible. The gelatinized state is expected to have the highest digestibility. Waxy rice starch, when treated under different high pressure conditions, underwent microstructural and morphological changes that altered its digestibility properties. At 600 MPa, the RDS starch, and at 400 MPa, the SDS starch, reached their highest digestibility respectively [22]. When similar tests at a pressure range of 120 MPa to 600 MPa were carried out on sorghum starch, the RDS successively reduced, and SDS and RS levels successively increased with pressure [25]. The glycemic index value is another term in assessing foods to determine the rate of their digestibility. It is determined by estimation of the increase in blood glucose with time [31]. Even though potato starch cannot be fully gelatinized by pressures up to 600 MPa, applying 6 cyclic pressures of 400 MPa for 10 min each followed by retrogradation of 7 days produced starch that underwent lower in-vitro hydrolysis [33]. This would mean a slower release of glucose, leading to food of low glycemic value (a food that would lead to longer duration of fullness). Thus, the properties of the final product can be easily adjusted to the desired characteristics with the use of HPP by varying pressure, temperature and duration of treatment.

#### 2.1.5. Applications

Corn starch gains some unique properties after high pressure treatment. A study was conducted on high pressure treatment of corn starch and its use as a carrier for molecules of nutritional value [36,37]. Three types of treatments were conducted for comparison. Alkali treatment (with NaOH), HPP treatment (at 400 MPa for 35 min and an initial temperature of 38 °C) and combined alkali and pressure treatment were carried out. The microstructure analysis showed that all treatments increased the porosity of the starch granules. The alkali treatment produced higher numbers of pores with larger pore volume when compared to HPP treated corn starch. The combined treatment produced many macropores and hence, a higher pore volume than native starch. Structure analysis showed that when zinc and magnesium were loaded into the granules, the alkali treatment proved to be a better method than HPP. The combined treatment did not act synergistically and gave moderate results. Since the microstructure analysis proved to be positive for the combined treatment, further study is required. Trials using other nutrients with different molecular sizes may be carried out. High pressure treated corn starches have also shown enhanced emulsion stabilization properties. During the homogenization process of sunflower oil mixed with the treated corn starch, the starch disintegrated [29]. The broken fragments of starch contributed to the stabilization of the oil droplets. The emulsion stability increased with the concentration of the starch. The creaming stability also increased with the concentration of the starch. The addition of NaCl to the process allowed the formation of droplets of larger size in the emulsion. The presence of NaCl did not affect the flow behaviour of the emulsion but the creaming stability was significantly reduced.

HPP gelatinization has been used in manufacturing foods and studies have shown both positive and negative impact on food quality. HPP on cake batters did not give any favourable results [38]. The cake made from the pressure treated batter had traits such as less expansion on baking, darker crust colour and much harder texture. It was stated that for a good quality cake, a higher gelatinization temperature is more desirable. In addition, a lower batter density provides a greater exchange area for the rise during baking. Since HPP reduced the gelatinization temperature and the volume during treatment, the process imparted undesirable characteristics to the cake. A method for developing resistant starch by treating the B Type starch was developed by making use of HPP treatment [39]. The technique involved mixing B type starch with water in the ratio 1:2 up to 1:10, vacuum packing in nylon-polyethylene bags and treatment at a pressure of 100–600 MPa at 20–50 °C. Since this method did not involve the use of any enzyme or reagents in the manufacturing process, it was claimed to have a ‘green’ cleaning process. The gelatinization using HPP at >400 MPa was uniquely used to produce foods with granular pregelatinized starch. Certain pressure-sensitive starches can be added to foods that are hermetically sealed and then HPP treatment can be conducted to get shelf-stable food products. This was carried out in another study, where the starch was used in the process to create fat replacements, thereby reducing the calorie content of the foods [40]. This process is advantageous because digestibility of starch is closely linked to the degree of gelatinization and the extent of gelatinization can be easily controlled by adjusting the processing parameters of pressure, temperature and duration.

From an industrial perspective, the initial study of setting the processing variables becomes necessary to achieve the levels of gelatinization or retrogradation while reviewing viable methods of commercialization. One such tool was presented by determining state diagrams of potato starch [41]. The state diagrams showed treatment pressure versus starch content graphs enabling the classification of processed starch into: completely gelatinized, partially gelatinized, completely gelatinized with retrogradation and partially gelatinized with retrogradation.

### 2.2. Gelatinization of Collagen

**Figure 6:**
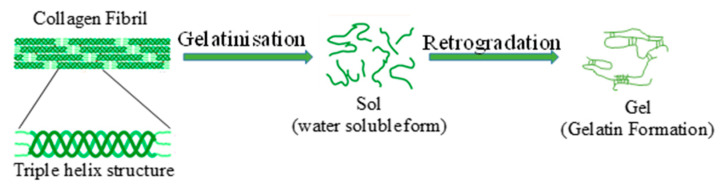
Gelatin made from collagen gelatinization (redrawn from von Endt and Baker 1991 [45]).

Gelatinization is not only restricted to starch. Gelatin or gelatine, commonly used as a gelling agent in foods, is made from the collagen derived from animals. Collagen is a triple-helical heterotrimer and is stabilized by hydrogen bonds [42]. Collagen is treated with dilute acid or alkali for several days so that the hydrogen bonds and other interlinks present in the coils are cleaved such that water-soluble gelatin is produced. This is gelatin or gelatinized collagen (Figure 6). There have also been a few studies on the gelatinization of collagen using HPP. Use of HPP in gelatinization of collagen with acid as the pressure transmitting medium dramatically reduces the processing time. An investigation compared the traditional method of acid treatment with the high pressure method [42]. Results showed that HPP gelatinization carried out at 500 MPa reduced the processing time from more than 26 h to 15 min. Though the pressure treated collagen had a comparatively lower yield than the traditional method, the physical properties of the HPP treated gels were superior. The gel strength and rheological properties of high pressure processed gelatins were better than the traditionally prepared gelatins. The hardness, gumminess, chewiness and adhesiveness of the jelly made from HPP treated gelatin were greater than the commercial gelatin, while cohesiveness and springiness were similar [43]. Increasing acid strength for extraction increases the yield but slightly reduces gel strength [44].

## 3. Forced Water Absorption in Foods Using HPP

**Figure 7:**
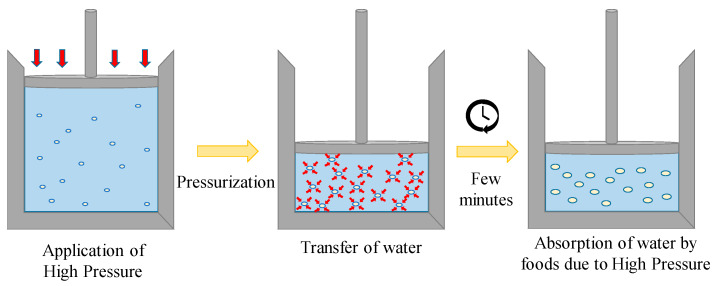
Representation of forced water absorption by foods under high pressure.

Starch is one of the primary components in foods, but there has been some research on the effect of HPP treatment on the properties of whole foods as well. The process of immersion of foods into liquids for softening is called soaking. The process of soaking is generally long, and HPP has been used to fasten the process (Figure 7). High pressure conditions can change many physico-chemical properties of foods such as the changes in diffusivities, nutritional and anti-nutritional compound levels, etc.

**Figure 8:**
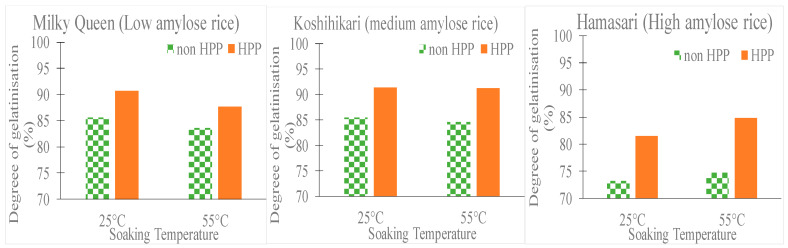
Comparison of the degree of gelatinization after cooking of three varieties of rice with different levels of amylose with their HPP treated counterparts (plotted using the data from Yamakura et al., 2005 [47]).

When whole grain glutinous rice was subjected to HPP, it was observed that the diffusion coefficient of water increased with temperature up to 300 MPa, after which temperature had no significant effect [46]. The effective diffusion coefficient of water also increased from 20 °C to 50 °C after which it decreased. The explanation stated for this phenomenon was the occurrence of gelatinization. The gelatinization of the rice granules restricts the transport (diffusion) of water. However, irrespective of the change in the diffusion coefficient, the quantity of water absorbed by the rice and the rate of its absorption increased with pressure and temperature. The effect of presoaking for 30 min, followed by high pressure treatment at 400 MPa for 10 min and final soaking overnight led to a rice variety that had enhanced properties [47]. The HPP processed rice had higher digestibility and starch with higher swelling capabilities. HPP led to a higher degree of water penetration in the rice, thus causing a higher level of gelatinization to the starch (Figure 8). The rice was claimed to have more palatable properties. A similar study conducted by Zhu et al. [48], recommended the combination of HPP and partial milling of brown rice to enhance its processing efficiency and quality. Foxtail millet, a type of cereal grain, was subjected to long durations of HPP [49]. There was a drop in the effective diffusion coefficient observed above 200 MPa for germinated and 600 MPa for non-germinated grains. The degree of gelatinization and water uptake increased with pressure, temperature and time. In addition, the nutritional components such as antioxidants and total phenolic activity were enhanced, whereas anti-nutritional compounds such as tannin and phytic acid decreased with high pressure.

**Figure 9:**
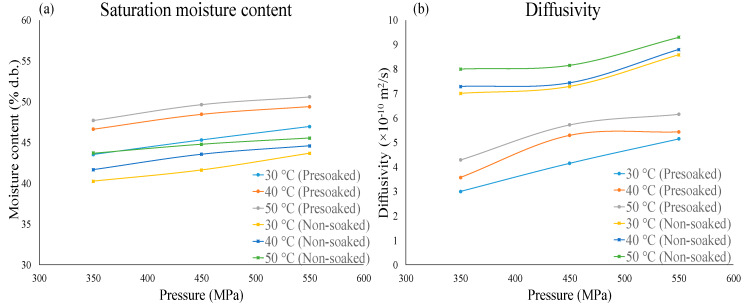
(**a**) Water uptake and (**b**) water diffusivity in presoaked and nonsoaked paddy grains subjected to HPP (plotted using the data from Ravichandran, Purohit and Rao, 2018 [50]).

In another study, paddy grains of the Basmati variety were subjected to HPP with pressure, temperature and time in the range of 350–550 MPa, 30–50 °C and 5–30 min respectively [50]. This was done to establish the water absorption and gelatinization kinetics. The effect of presoaking was also studied. The non-soaked grains showed a higher rate of water uptake, whereas the presoaked grains showed a higher degree of gelatinization with the maximum at the highest level of pressure and temperature studied, i.e., 500 MPa, 50 °C. Temperature and pressure were shown to have a synergistic action on water absorption. Results showed that under the influence of high pressure, the water absorption and diffusivity were much higher compared to grains soaked at atmospheric pressure. Presoaked grains exhibited a higher saturation moisture content, whereas the non-soaked grains showed higher diffusivities (Figure 9). A first-order kinetic model was used to describe gelatinization. Based on the energy of activation (E_a_) and activation volume (ΔV), adverse effects of pressure on gelatinization was observed at extreme high pressure. Ravichandran et al. [50] further explained that the progressing of gelatinization depends on the first order rate constant (*k*). *k* is related to E_a_ and ΔV by the Arrhenius and Eyring equations respectively. An increase in the value of E_a_ leads to a subsequent decrease in *k*. In thermal gelatinization, the value of E_a_ decreases with increase in temperatures above 60 °C. This eases the process of gelatinization at high temperatures. In pressure gelatinization, an increase in pressure leads to the rise in E_a_. The ease of gelatinization is hence lower. This is because an increase in pressure restricts starch granule swelling. Negative values of ΔV were observed which indicated the sensitivity of the grains to pressure. This meant the occurance of compression of grains during pressure treatment. The negative value of ΔV further sustained the explanation of the restriction of starch granule swelling due to pressure. Hence, a lower pressure of 450 MPa for treatment was suggested. The conclusion of the study was that this method of using HPP was a better alternative to thermal soaking because it reduces soaking time and also induces partial gelatinization in the paddy grains.

Dried beans were initially soaked and then treated with a high pressure of 600 MPa for different durations. This was then compared with thermally treated (HT) (95 °C) and high pressure/high temperature process (HPHT) (600 MPa, 95 °C) [51]. Softening of the beans is an important process before eating. This is because raw beans are too hard to be disintegrated by mere chewing and the softness is an indicator of the suitability for consumption. The results showed that the high pressure process alone was not able to reduce the hardness of the bean even after long processing times. The presence of heat with or without pressure is necessary to soften the bean. This was attributed to thermal pectin solubilization. Although the HPHT was found to have a higher degree of starch hydrolysis than HT samples, the process did not impart enough enhancement of any property to make the process economically viable.

HPP has been used to treat cut pieces of tubers and the process has been shown to alter some of the properties. When potato discs were subjected to moderate heat and pressure treatment (MHPT) (75–95 °C, 100 MPa), the firmness increased at the temperature range of 75–85 °C. MHPT also slightly increased tissue sloughing while undergoing colour change similar to that of heat treatment [52]. Amylase acts on gelatinized starch and causes saccharification of the starch. Sweet potatoes contain internal amylases. If the starch present in sweet potato is gelatinized, the internal amylases can cause saccharification and this would give a higher yield of reduced sugars in extraction. However, the internal amylases are inactivated at high temperatures near 100 °C. An experiment on using HPP to cause gelatinization in sweet potato was carried out [53]. Treatments were conducted at different levels of pressure and temperatures for different durations. The process did not affect the amylase activity, but the reducing sugars extracted from the pressure treated samples were similar or slightly lesser in quantity than that extracted from untreated sweet potato pieces. This indicated insufficient gelatinization for saccharification. The process of HPP showed an inhibitory effect on the gelatinization and/or saccharification at lower pressures of 100 MPa and 200 MPa. The reduced sugar content in the samples treated at high pressures of 500 MPa and 70 °C were comparable, but were still lower than those treated at atmospheric pressure at 80 °C for the same duration. The reason behind this anomaly of the initial fall in the reducing sugar content with pressure remained unclear.

## 4. Pressure Assisted Infusion in Foods

When the process of soaking (carried out at atmospheric pressure) is used to transfer some compounds of value to foods, the transfer occurs through the process of osmosis, where there are two components; the solvent (usually water) and the solute (the component intended for transfer). The concentration of solute in the soaking solution can be increased only up to a critical concentration. On exceeding that concentration, the moisture gets driven out, dehydrating the food that is not generally desirable. In such situations, high hydrostatic pressures can be used to force the transfer of the food value compounds.

Using high pressure processing of foods can osmotically induce a mass transfer process to drive certain compounds of value into or out of foods according to the requirements. The diffusion of both the solvent and solute happens simultaneously during soaking, and the directions of diffusion can be the same or opposite depending on the osmotic potentials. This feature can be effectively made use of to increase the quality of foods in terms of nutritional content and flavour enhancement. If a food item is placed in a nutritive medium followed by HPP of the sample, the nutrients can be driven into the food while maintaining the moisture in them. The mechanism for the transfer is not well understood. A research on HPP treatment of brown rice suggested that HPP creates some microfractures on the surfaces of grain, facilitating water transport [54]. This can be taken as a plausible explanation for the transport of nutrients along with water. However, this is a low moisture content grain and developing cracks cannot be the mechanism for high moisture foods. When high moisture fruits were processed for nutrient transfer using HPP, two explanations have been hypothesized for the mechanism of transfer. High pressure conditions affect the cell structures of fruits or vegetables, thereby affecting their permeability and allowing them to uptake nutritional compounds from the surrounding medium [55,56]. However, for quercetin-infused cranberries, Mahadevan et al. [57] reported that even though the cell permeabilization does play some role in increasing the transfer, this effect is not dominant. Osmosis has been stated to be mainly responsible for the diffusion. This conclusion was supported by showing a similar degree of cell permeability in samples before and after HPP. One interesting point to note in this experiment is that the cranberries were impermeable to quercetin initially and had to be scarified before HPP treatment. This shows that, regardless of whether cell permeabilization is dominant or not, cell permeabilization is necessary for nutrient transfer. HPP can create cell ruptures in some foods while in others, external scarification may be necessary. Overall, the process of HPP preserves the main solid matrix of foods when transferring components to different phases.

### 4.1. Aroma Infusion

**Figure 10:**
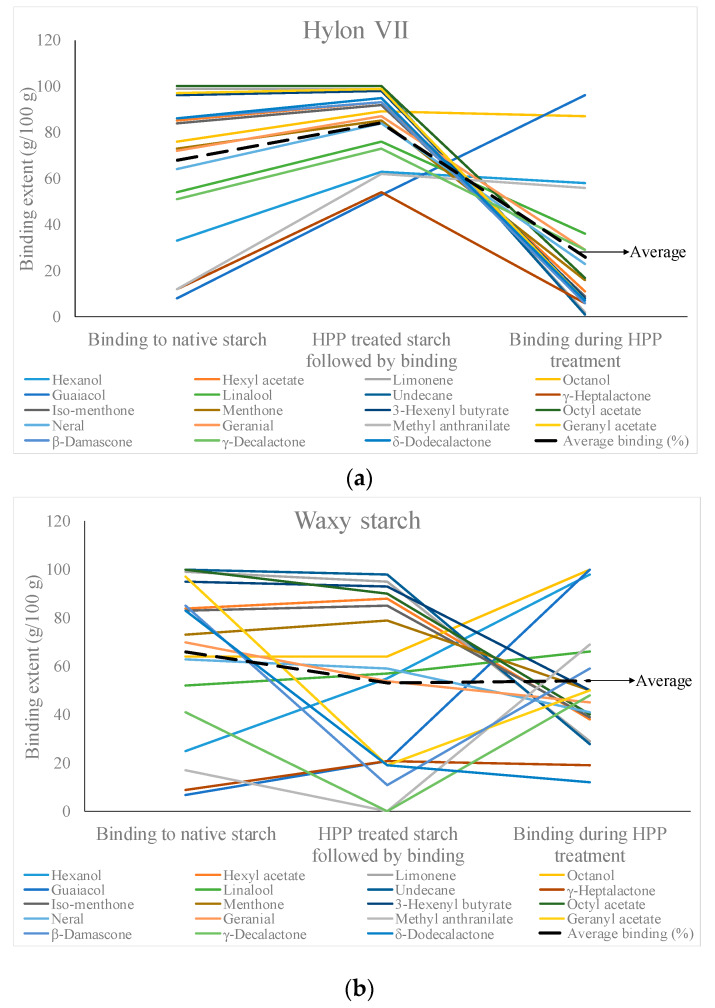
Comparison of binding properties of different odorants to (**a**) high amylose maize starch (Hylon VII), (**b**) no amylose maize starch (waxy starch) treated in various ways (plotted using the data from Błaszczak, Misharina, Yuryev, and Fornal, 2007 [58]).

HPP has had some application in binding different aromas to foods. Some common odorants were mixed with maize starch in native form, with HPP treated maize starch and with maize starch during HPP treatment at 650 MPa for 9 min [58]. Different maize starches with varying amylose content were studied for comparison. The binding extent of these different methods was then analyzed. Hylon VII, a high amylose maize starch, showed similar or slightly enhanced binding characteristics after it was treated with HPP (Figure 10a). However, when it was mixed with odorants during HPP, the binding was poorer for most varieties of the odorants excepting hexanol, guaiacol, methyl anthranilate, and octanol. Guaiacol and methyl anthranilate had very poor binding characteristics for native starch. In waxy maize starch with no amylose, there were a few compounds whose sorption reduced in the HPP treated starch. Although the overall average sorption of odorants reduced for both type of HPP treatments, hexanol, guaiacol, methyl anthranilate, and octanol, along with a few more compounds, showed enhanced sorption characteristics during HPP treatment (Figure 10b). The process of HPP also led to gelatinization of starch and was inversely related to the amylose content.

### 4.2. High Pressure Impregnation (HPI)

When any compound is used in the liquid form or in a dissolved form for infusion by pressure into a solid food (generally a solid porous matrix), it has been referred to as high pressure impregnation or HPI. Vatankhah and Ramaswamy [59] used HPI for the impregnation of ascorbic acid in apple cubes. They found the ascorbic acid content in the apple pieces increased with pressure. They also checked the effect of viscosity in HPI by making use of different concentrations of chitosan. The result showed a decrease in the level of mass intake of chitosan with an increase in its concentration. The impregnation of chitosan also increased the pressure stability of the apple, which would otherwise collapse under high pressure due to the presence of small air pockets. Osmotic dehydration (OD) was compared with vacuum impregnation (VI) and high pressure impregnation by adding sucrose and calcium lactate in unripened and ripened mango chunks [60]. All samples after OD, VI or HPI were subjected to further OD for up to 4 h before the examination. The effect of the presence of pectin methylesterase (PME), which is generally used for improving food firmness, was also evaluated. It was found that both VI and HPI led to an increase in the soluble solids gain and lower water loss in comparison to OD alone. HPI, however, had lesser pronounced effects than VI. The presence of PME for all cases led to a slight increase in firmness and work of shear. The soluble solids gain, with or without the presence of PME, increased in the unripened mango but decreased in ripened mangoes. This was because of two reasons; the sturdy cellular structure of unripened mangoes and the lower sugar content in them. When water leaves the fruit tissue, the susceptibility of the tissues structure to collapse in unripened mangoes is much lower than ripened ones. The collapse of tissue structure can lead to physical hindrance of the osmotic solution from entering the fruit. In addition, lower sugar content in unripened mangoes results in a greater concentration gradient with the osmotic solution, thereby promoting a higher soluble solids gain. PME-pretreated baby carrots with HPP treatment with calcium lactate gluconate (CLG) showed that the high pressures increased their calcium content by almost seven times [61]. The HPP-treated baby carrots had calcium contents of more than three times that obtained by soaking at atmospheric pressure or with vacuum infusion techniques. Experiments using Box-Behnken design showed that the pressure level, processing time and CLG concentration are directly related to the increase in the calcium content and hardness of the carrots. The application of cyclic pressures also causes a significant increase in calcium. The follow-up study using SEM imaging showed that HPP caused cell damage in the carrots [62]. The extraction of beta carotene from the carrots increased by three to five times in comparison to the untreated variety. The effects of individual processing variables, i.e., pressure, processing time, CLG concentration and PME pretreatment on the amount of beta carotene extracted were not significant. HPP also led to a mild to moderate darkening of the carrots after infusion. 

Another study using HPI was done to impregnate oil into selected fruits through water-based emulsions [63]. Oil and Tween 80, an edible emulsifier, were used in different ratios to form different emulsified oil mixtures. Twenty per cent of these emulsified oil mixtures were homogenized along with 80% water to form the infusate. It was found that the presence of hydrophilic emulsifiers in the solutions facilitated the transport of oil into the inner layers. Without the presence of emulsifiers, oil transfer in fruits was not very efficient. In addition, the higher the concentration of the emulsifier, the lower the transport, whereas higher oil concentration gave higher transfer. This method could prove to be a very efficient method for the fortification of foods with lipid-soluble vitamins, such as vitamin A, vitamin K, etc. in foods [63].

A unique study involving a combination of high pressure gelatinization and high pressure infusion was carried out with paddy rice [64]. Rice loses most of its thiamine, present in the bran and germ layers, during its processing from brown rice to white rice. Parboiled rice, however, has much of the thiamine retained in it. Rice undergoes gelatinization during parboiling and gelatinization facilitates the process of thiamine transfer. Since high pressure environments have shown to cause gelatinization, paddy rice was subjected to pressures of 450 MPa and 600 MPa at the temperatures of 50 °C and 70 °C for 15 min and 30 min. The results showed that HPP caused a higher transfer of thiamine as the pressure and temperature increased. Long processing times, however, led to its degradation. The HPP treated rice maintained the grain dimensions whilst parboiling rendered the rice shorter and broader.

### 4.3. Flavour Infusion

**Figure 11:**
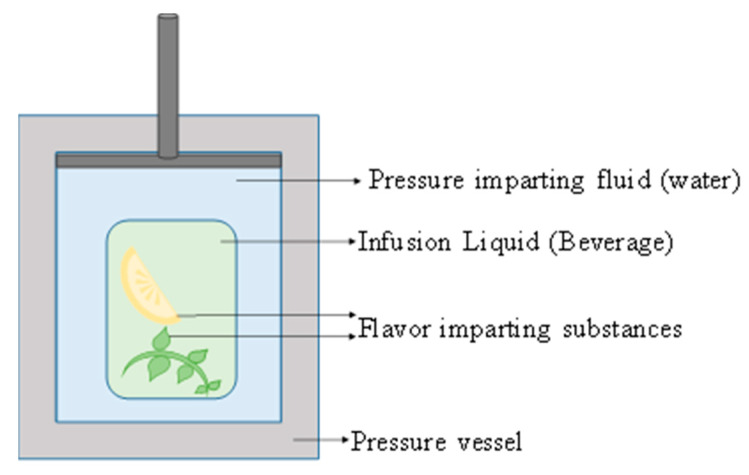
High pressure infusion (HPI) of flavour into beverages (redrawn from U.S. Patent No. 15/975,989, 2018 [67]).

HPP has been used to impart flavours to foods. Villacís, Rastogi, and Balasubramaniam [65] demonstrated the use of HPP for adding salt and moisture to turkey breast while improving its textural properties. Lemus-Mondaca et al. [66] also used HPP to impregnate salt and water into jumbo squid to enhance sensory characteristics. The Weibull model was reported as the best fit for the mass transfer of compounds into the jumbo squid. When the flavour from solid foods like lemon needs to be infused into any drink, the flavour imparting substance must be placed in the target liquid. On the application of high pressure, the flavour components will be transferred to the drink. The brewing of coffee or tea needs to be done at high temperatures. Cold brewed beverages take considerably longer times to reach similar infusion levels. If a pot of hot brewed coffee takes five to ten minutes for preparation, the cold brewed variety can take several hours [67]. Thus, HPP can make the infusion process faster and much more effective. Hence, a design for the infusion of alcoholic beverages with at least 20% alcohol content was carried out with the HPP technology, as shown in Figure 11 [67]. This technology can be effectively applied in the near future for the enhancement of flavours in the beverage industry.

## 5. Conclusions

High pressure processing has many unexplored possibilities for food quality improvements. A significant amount of work has been done in the area of high pressure gelatinization but with limited industrial-scale applications. The niche market for high pressure infusions and high pressure impregnations is an upcoming method, leading to a high potential for food quality enhancements. The ability to make use of the three parameters of pressure, temperature and time can be optimized for the design of food products with specific properties. Since HPP also inactivates most pathogens, it can be very useful for the production of final packaged ready-to-consume foods that undergo gelatinization and/or infusion during pressure treatment. The impregnation of vital nutrients such as vitamins, flavours and stabilizers into foods are other beneficial applications of this method. Although aroma binding to foods is an innovative idea for augmenting the sensory attributes of foods, the application of HPP in this field has not been extensively studied yet. The simultaneous quality enrichment, along with microbial inactivation, would be very useful in developing processed foods and making the overall expensive process of HPP viable.

## Figures and Tables

**Table 1:** Important highlights of the studies on different HPP treated starches.

Type of Starch	Pressure	Temperature	Time	Property Assessed	Outcome	Reference
Tapioca	600 MPa	30–80 °C	10–30 min	Hardness Post storage changes in hardness Comparison with thermally treated starch	Harder gels at low temperatures. Long treatment led to a slight decrease in hardness Higher starch-starch and starch water interactions Better storage efficiency	Vittadini et al. [18]
Wheat	600 MPa	25 °C	15 min	Hardness Retrogradation rate Comparison with thermally treated starch	Greater hardness in gels Lower rate of retrogradation	Douzals et al. [19]
Wheat	300–600 MPa	25 °C	10 min	Hardness Gumminess Chewiness Comparison with thermally treated starch	Hardness, gumminess and chewiness were high only till gelatinization <40%	Liu et al. [16]
Potato	400 MPa (3 cycles) followed by 600 MPa (6 cycles)	21 °C	Each cycle 10 min	Cyclic pressure treatments Post retrogradation analysis	Eruptions, abrasions and disruptions of starch granules Highly compact gel Post retrogradation, gel was more compact and dense	Colussi et al. [20]
Maize	300 MPa	20 °C	5–60 min	Moisture adsorption-desorption analysis	Upward shift of the MSI Ability to higher moisture at the same water activity	Santos et al. [21]
Waxy rice	100–600 MPa	25 °C	20 min	Lamellar repeat distance in starch granules Crystallinity	With increase in pressure Lamellar repeat distance increased Crystallinity decreased	Li et al. [27]
Chestnut	400–600 MPa	35–38 °C	10 min	Degree of gelatinization Granule particle size or hydration properties	Pressure resistant No change in granule particle size or hydration properties	Ahmed et al. [23]
Sorghum	120–600 MPa	Room temperature	20 min	Gelatinization Water absorption capacity and thermal stability Oil absorption capacity, swelling power and viscosity	B type crystalline pattern at 600 MPa Increase in water absorption capacity and thermal stability Decrease in oil absorption capacity, swelling power and viscosity	Liu et al. [25]
Mango kernel	300–600 MPa	38 °C	10 min	Retrogradation Structural analysis of the starch granules	Lower aggregation and retrogradation tendencies Insignificant structural changes in the starch granules, however significant changes in the smoothness of the granule surfaces	Kaur et al. [26]
